# Incidence of Symptomatic Venous Thromboembolism in Patients with Hemophilia Undergoing Hip and Knee Joint Replacement without Chemoprophylaxis: A Retrospective Study

**DOI:** 10.1111/os.12444

**Published:** 2019-04-01

**Authors:** Hui‐ming Peng, Long‐chao Wang, Ji‐liang Zhai, Chao Jiang, Xi‐sheng Weng, Bin Feng, Na Gao

**Affiliations:** ^1^ Department of Orthopaedic Surgery, Peking Union Medical College Hospital Chinese Academy of Medical Science and Peking Union Medical College Beijing China

**Keywords:** Blood coagulation disorders, Blood coagulation factors, Thrombosis, Total hip arthroplasty, Total knee arthroplasty

## Abstract

**Objective:**

To establish the prevalence of clinically significant venous thromboembolic events (VTE) in hemophilia patients undergoing total hip arthroplasty (THA) and total knee arthroplasty (TKA) without chemoprophylaxis and a modified coagulation factor substitution.

**Methods:**

A cohort of patients who underwent THA and TKA from June 2002 to April 2017 were included. Based on World Federation of Hemophilia (WFH) guidelines, a modified coagulation factor substitution regimen was adopted. All patients were under a standardized postoperative protocol with routine mechanical prophylaxis against VTE. None of the patients received prophylactic anticoagulation. Only symptomatic patients were referred for radiological examination to exclude VTE. We evaluated the patient demographics and calculated the prevalence of VTE in our cohort.

**Results:**

A total of 98 patients were reviewed. The patients were all men. Thirty‐one patients underwent primary THA with 39 hip arthroplasties (only 1 case with hemophilia B) and 67 patients underwent primary TKA with 101 knee arthroplasties (5 cases with hemophilia B). The mean age was 34.2 ± 7.8 years. The mean body mass index was 21.2 ± 5.7 kg/m^2^. There was 100% compliance to mechanical prophylaxis. The mean time to ambulation was 6.8 days (±2.5 days), and the mean hospital stay was 32.4 days (±7.1 days). There was only 1 hemophilia B patient with clinically significant VTE. None of the other 97 surgical cases had symptomatic VTE within 6 months after the procedure. This translates to a prevalence of 1.02%.

**Conclusion:**

Given the low incidence (1.02%) of clinically significant VTE in our cohort, routine chemoprophylaxis in hemophilia patients undergoing THA and TKA may not be needed.

## Introduction

Hemophilia is an inherited bleeding disorder due to a deficiency of factor VIII (hemophilia A) or factor IX (hemophilia B). In this group of patients, recurrent hemarthroses result in hemophilic arthropathy (HA) and it is a common and occasionally inevitable complication that affects greater than 90% of patients with hemophilia before the age of 30 years. The pathogenesis of HA begins with hemophilic synovitis induced by recurrent hemarthrosis, followed by joint erosion with cartilage damage and erosion of adjacent bones. The main target joints are hips and knees^1^. Surgery is the most effective, thorough treatment for hemophilic destructive osteoarthropathy. Due to access to safe factor replacement products, the number of patients with hemophilia undergoing total hip arthroplasty (THA)/total knee arthroplasty (TKA) has also been increasing^2^, ^3^, ^4^. There are multiple challenges, however, such as atypical anatomy, abnormal bone structures, osteoporosis, and bone defects. The complication rates are high, including infection, inhibitor formation, prosthesis failure, and perioperative bleeding. The concern regarding bleeding complications in this special population has led to varying practice in perioperative management.

Deep venous thrombosis (DVT) and pulmonary embolism (venous thromboembolism [VTE]) are well‐known complications after TKA and THA^5^. In individuals who do not have hemophilia, a 40%–60% chance of asymptomatic deep vein thrombosis (DVT) and 2%–5% chance of symptomatic VTE after hip replacement have been described. After a total knee replacement, the risk of DVT can be even higher than hip replacement (40%–85%)^5^. Therefore, routine thromboprophylaxis has been recommended for hip and knee arthroplasties in guidelines^5^, ^6^.

Patients with hemophilia are at low risk for thromboembolic complications because of the coagulation factor deficiency^7^. However, correction of the hemostatic defect through perioperative use of coagulation factor concentrates theoretically increases their risk of VTE such that it is similar to the risk in the general population. Therefore, chemoprophylaxis for hemophilia patients undergoing hip and knee arthroplasties remains controversial^8^. In contrast, perioperative clotting factor concentrate replacement is administered to correct the inherent hemostatic defect in persons with hemophilia, potentially rendering these patients at risk of developing VTE postoperatively^9^. Persistently elevated levels of FVIII are a risk factor for thrombosis in the general population^10^. In developing countries such as China, due to patients’ economic difficulties and limited sources of coagulation factor, we modified the coagulation factor substitution regimen based on the World Federation of Hemophilia (WFH) guidelines. According to our modified protocol, the factor replacement consumption per individual patient seems to be less than that of other hemophilia centers.

We assume that the reduction in the amount of factor replacement usage and lack of chemoprophylaxis at the same time may reduce the risk of VTE while reducing the economic burden of patients.

Do hemophilia patients with our modified coagulation factor substitution truly have a lower incidence of VTE following THA and TKA? Is there a need for routine chemoprophylaxis following THA and TKA in this group of patients?

This retrospective cohort study seeks to find answers to the above questions. The purpose was to follow up patients who underwent THA and TKA for HA and to evaluate the VTE complications associated with it. Herein, we review our institutional experience with outcomes of TKA and THA in patients with hemophilia, with our study comprising the largest collective in the current literature to our knowledge.

## Materials and Methods

This retrospective study was approved by the Peking Union Medical College Hospital Institutional Review Board (S‐k201).

### *Participants*

We reviewed the institutional medical records of all patients with hemophilia A or B enrolled in the orthopaedic department at the Peking Union Medical College Hospital who underwent THA and TKA from 1 June 2002 to 30 April 2017. Criteria for diagnosis and classification of the severity of hemophilia conformed to the recommendations of the Scientific Standardization Committee of the International Society on Thrombosis and Haemostasis^11^.

Exclusion criteria were: (i) a history of pulmonary embolism (PE) or DVT in the previous year, a history of varicose veins, and/or chronic venous insufficiency; and (ii) records being incomplete.

### *Interventions*

##### *Total Hip Arthroplasty*

A posterolateral approach was used, and the surgical technique was similar to the standard THA procedure. However, we encountered more challenges and required some special care. Hypertrophic synovium, hemosiderin, and the joint capsule should be thoroughly resected to reduce the rate of bleeding. Release of soft tissue may be required for most of the patients because soft tissue contracture was common in hemophilic arthropathy. No drainage was regularly used in THA.

##### *Total Knee Arthroplasty*

A tourniquet was used in all cases. A standard midline incision was used and a medial parapatellar arthrotomy was performed. Extensive removal of the synovium was performed. We preferred to release the posterior capsule and soft tissue rather than perform an additional osteotomy for minor deformities. For patients with severe flexion contracture, initial additional osteotomy of the distal femur was performed, and additional osteotomy of the proximal tibia was performed. No patient received patella resurfacing with prosthesis. Tranexamic acid (TXA, 1000 mg) was intravenously administered to all patients 15 min before skin incision, and topical TXA solution (1000 mg) was applied intra‐articularly to each operated joint approximately 5 min before closure. We performed patelloplasty for all the cases involving articular surface smoothing, osteophyte removal, and patellar rim denervation.

Operation duration was recorded from the beginning of skin incision to the finish of skin closure. For postoperative pain management, our standard protocol was application with PCA in the first 3 days, following oral NSAID on time and opioids on demand for next 2–6 weeks.

#### *Venous Thromboembolic Event Prophylaxis, Factor Concentrate Infusions, and Factor Activity*

The concentration of plasma FVIII and FIX was measured preoperatively to determine the strategy for perioperative coagulation factor substitution. Patients with hemophilia A were treated with plasma‐derived or recombinant FVIII, or recombinant FVIII, while those with hemophilia B were treated with activated prothrombin complex concentrate (APCC). The coagulation factors were infused as a single bolus 1 h before the surgery, with an additional bolus given if the operation was >6 h. The concentration of plasma coagulation factors and inhibitors was monitored on postoperative day (POD) 1, 4, and 7. Based on the WFH guidelines, we modified the coagulation factor substitution regimen due to the patients’ financial difficulties and limited sources of coagulation factor. The dosage of coagulation factor was adjusted correspondingly to maintain the peak level of factor concentration at: 80%–100% on the day of surgery; 60% at POD 1, 2, and 3; 40% at POD 4, 5, and 6; and 20%–30% thereafter. The substitution of coagulation factors was maintained until suture removal, which was done at 2–3 weeks postoperatively. Additional coagulation factors, an antifibrinolytic agent, or a blood transfusion was administered, depending on the swelling of soft tissues in the operative field, the amount of postoperative drainage, and the level of hemoglobin in the blood. Patients who were positive for coagulation factor inhibitors were treated with APCC or recombinant FVII.

We applied DVT/compression stockings as mechanical prophylaxis methods for all patients.

The surgeon and his team reviewed the patients daily, and any clinical signs of VTE were recorded in the case sheets. Only symptomatic patients with clinical signs of VTE were referred for radiologic and/or biochemical evaluation. Postoperatively, patients were followed up at: 2 weeks; 1, 3, and 6 months; and 1 year. Up until 6 months postoperatively, patients were asked about clinical symptoms of DVT, such as calf swelling, erythema, tenderness, and fever. The surgeon also examined each patient for clinical signs of DVT.

### *Comparison*

Because of the special population of the study as well as the very low prevalence of VTE, no comparative analysis was done.

### *Outcomes*

Patient data, including age at surgery, type and severity of hemophilia, the joint, and the type of surgery (primary *vs* revision arthroplasty), were abstracted. We also recorded the type of factor replacement, the factor levels, and the use (or lack thereof) of VTE prophylaxis (with stockings or without stockings). Operative reports, hospital summaries, and subsequent visit notes were reviewed to determine the occurrence of symptomatic VTE (DVT and pulmonary embolism) during hospitalization up to 6 months postoperatively.

### *Statistical Analysis*

Parameters assessed include age, race, gender, body mass index, type of anesthesia, tourniquet time, and number of comorbidities. Hypertension, diabetes mellitus, and asthma were the most common comorbidities in these patients. The prevalence of VTE in the patients was then calculated using a standard formula.

## Results

### *Patient Characteristics*

None of the patients were excluded from this study based on the above exclusion criteria, leaving a total of 98 patients for review. In our study group, the patients were all men. Thirty‐one patients underwent primary THA, with 39 hip arthroplasties (only 1 case with hemophilia B) and 67 cases underwent primary TKA with 101 knee arthroplasties (5 cases with hemophilia B). The mean age (and standard deviation) was 34.2 (±7.8) years. The mean body mass index was 21.2 kg/m^2^ (±5.7 kg/m^2^). All our patients underwent general anesthesia. All our patients used mechanical prophylaxis from the immediate postoperative period until discharge. The mean time to ambulation was 6.8 days (±2.5 days) and the mean hospital stay was 32.4 days (±7.1 days).

The average number of days of factor replacement was 16.78 (range, 14–25). Serum mean FVIII level was 74.07%. None was detected with positive coagulation factor inhibitor. The average FVIII consumption for operation and in‐hospital rehabilitation was 62228 IU.

### *Venous Thromboembolic Event Outcomes*

Only one case suffered symptomatic DVT (left deep femoral vein). This was a 29‐year‐old male patient with mild hemophilia B (baseline factor IX activity 10%) who underwent right THA for end‐stage hemophilia arthritis. APCC replacement was continued at 4000 daily. Symptomatic DVT (left deep femoral vein) was diagnosed by compression venous duplex ultrasound on POD 13. Elevation of the leg and anticoagulation with therapeutic dosing of low‐molecular weight heparin (LMWH) were conducted every 12 h. LMWH was discontinued 11 days after anticoagulation and swelling of the left lower limb resolved 24 days postoperatively. The incidence was 1.02%, as calculated by the number of patients, and 0.7% by the number of procedures.

A review of the case notes of all the other patients found that none had developed clinical signs and symptoms of VTE up to 6 months after surgery. One patient had to be readmitted for infection and underwent revision surgery. However, ultrasound assessment excluded the presence of any VTE in the patient.

### *Complications*

#### *Total Hip Arthroplasty Group*

No hematoma occurred in the hip. One patient complained of hyperalgesia on the dorsum of the foot on postoperative day 1 with no motor symptoms; this resolved spontaneously 2 days later. This patient also had right ankle swelling and blisters on postoperative day 6; this resolved after infusing higher doses of coagulation factor and with physiotherapy. Another patient suffered a proximal femoral shaft fracture, intraoperatively, which was treated successfully with cerclage cables.

#### *Total Knee Arthroplasty Group*

One case developed hemarthrosis, while another case had severe valgus deformity developed with skin necrosis and common peroneal nerve palsy when valgus deformity was corrected from 30° to 0°. One patient was diagnosed with late infection one and a half years after primary TKA. The patient was treated successfully by one‐stage revision surgery.

## Discussion

For hip and knee end‐stage hemophilic arthropathy, THA and TKA is the effective choice for pain relief, re‐alignment, bleeding control and function recovery^12^, ^13^. The exact incidence of VTE after total joint arthroplasty in patients with hemophilia is still unknown, and no control trials comparing the use of pharmacologic prophylaxis versus placebo have been done or are likely to be done. Therefore, all hemophilia centers will base their practices on the current state of knowledge and expert opinions until more valuable evidence is available. To the best of our knowledge, our series includes a larger number of hip/knee joint arthroplasties than the other reports^14^, ^15^. The incidence of symptomatic VTE was 1 in 98 (1.02%) patients, which is lower than the incidence reported in the literature for patients without hemophilia undergoing the same surgical procedure in the absence of VTE prophylaxis^6^. The absence of thromboembolic complications in the other 139 arthroplasty procedures (97 patients) coincides with reports from other large series^2^, ^3^, ^16^.

Another analysis of pooled data from published series of hemophilia patients undergoing arthroplasty showed an estimated incidence of symptomatic VTE of 0.5%; however, considering the time span of the other study, the small sample size and different prophylaxis methods after surgery, the incidence data does not seem so valuable^14^.

Reported routine application of chemophylaxis in patients with hemophilia varies greatly and remains controversial among different centers. Hermans *et al.* conducted a survey of European hemophilia treatment centers that suggests pharmacologic VTE prophylaxis was used by more than half of respondents^17^. A survey in 2009 of US hemophilia centers found that 67% believed that patients with hemophilia undergoing joint replacement had high enough VTE risk to warrant some type of VTE prophylaxis, but only 37% indicated that they routinely provide prophylaxis for this purpose^18^. Thirty percent of respondents believed there was high risk of VTE but provided prophylaxis only for select patients (e.g. a high‐risk patient with coagulation factor levels 100%)^10^. Prophylaxis options included compression stockings (32%), sequential intermittent compression devices (35%), and pharmacologic agents (33%), such as LMWH, warfarin, unfractionated heparin fondaparinux, and aspirin (in decreasing order of frequency). Better risk stratification is needed to identify patients who would benefit from pharmacological prophylaxis.

Our study has revealed that only 1 patient with hemophilia B underwent THA without anticoagulation and suffered from symptomatic VTE. We did not examine the factor V Leiden in our study population but believe that protective genetic mechanisms may be an important reason for the low prevalence of VTE. It is reported in approximately 5% of Westerners but is less common in Africans and rare in Asians^19^, ^20^. Further detailed assessment of risk factors for symptomatic VTE was not possible in our practice because of the special population of the study as well as the very low prevalence.

It was reported that persistently elevated levels of FVIII were associated with thrombosis events in the common population^10^. However, there is lack of specific information about factor substitution strategy. This prompted us to more strongly consider the role of factor replacement on VTE prevention. The 2010 WFH guidelines recommended substitution therapy in which FVIII should be maintained at 120% preoperatively, at 60%–80% in the first 3 days postoperatively, and at 50% on the 4th–14th days postoperatively^21^. Due to the concern about hemophilia patients’ financial burden and limited sources of coagulation factor in China, we modified the coagulation factor substitution regimen based on the WFH guidelines. The dosage of coagulation factor was adjusted correspondingly to maintain the peak level of factor concentration at: 80%–100% on the day of surgery; 60% at POD 1, 2, and 3; 40% at POD 4, 5, and 6; and 20%–30% thereafter. Our hypothesis is that reduced usage of FVIII or APCC without chemoprophylaxis may lower the risk of VTE and postoperative bleeding events. Lower peak level of coagulation factor was focused on in our study. There were no uncontrolled recurrent joint bleeding or hematoma formation cases after THA and TKA in our cohort. This study may be able to provide a new perspective on how to prevent VTE in hemophilia patients who underwent THA and TKA. To optimize the balance between bleeding and thrombotic risk, our modified factor replacement protocols and monitoring seem to be helpful as well.

Mannucci *et al.* recommended LMWH 6–12 h after orthopaedic surgery. The data on the use of intravenous heparin in hemophilia are also limited because most patients had not had these high‐risk surgeries or cardiac interventions^22^. Only two case reports showed the safety of heparin use in patients who underwent cardiac catheterization or who received a therapeutic heparin dose due to VTE and their factor levels being above 100%^23^. However, routine anticoagulation is not without its risks.

These complications have a detrimental effect on postoperative rehabilitation, increase the consumption of coagulation factor replacement, and prolong hospitalization. These effects may place a strain on hospital and public health resources. The low incidence of VTE in our study necessitates a deeper risk benefit evaluation of routine chemoprophylaxis in patients undergoing THA and TKA.

The limitations of our study were related to its retrospective nature. Confounding and bias are inherent in a retrospective study despite efforts made to reduce their impact on error. Furthermore, due to the very low prevalence of VTE, statistical analysis was not feasible. For the same reason, comparative statistics were not possible. Our study also failed to identify any risk factors for VTE in hemophilia patients in terms of type of factor replacement, dosing, and evolution of surgical technique precluding firm recommendations. Although our series included the largest number of THA and TKA procedures, data have limited numbers and should be interpreted with caution. Hence, it is not appropriate that we make any recommendation about chemoprophylaxis against VTE in patients undergoing TKA. Additional prospective studies with larger sample sizes are needed in the future.

### *Conclusion*

The prevalence of clinically significant VTE in our patients who underwent THA and TKA without routine chemoprophylaxis is 1.02%. VTE is rare but can still happen; we discreetly recommend that conventional prophylactic anticoagulation is not routinely necessary. Anticoagulation prophylaxis should only be considered in patients with hemophilia with multiple high‐risk factors for thromboembolism. However, further studies with more patients included are needed.
